# Health seeking and access to care for children with suspected dengue in Cambodia: An ethnographic study

**DOI:** 10.1186/1471-2458-7-262

**Published:** 2007-09-24

**Authors:** Sokrin Khun, Lenore Manderson

**Affiliations:** 1 National Centre for Health Promotion, Ministry of Health, Phnom Penh, Cambodia; 2 School of Psychology, Psychiatry and Psychological Medicine, Faculty of Medicine, Nursing and Health Sciences, Monash University, Caulfield East, Victoria, 3145, Australia

## Abstract

**Background:**

The continuing contribution of dengue fever to the hospitalization and deaths in hospital of infants and small children in Cambodia is associated with delays in presentation for medical attention, diagnosis and appropriate care. It is important to identify the reasons that influence these delays, in order to develop appropriate interventions to redress the impact of dengue.

**Methods:**

Data on health seeking were collected during an ethnographic study conducted in two villages in the eastern province of Kampong Cham, Cambodia in 2004. Interviews were conducted with mothers whose children had been infected with suspected dengue fever, or who had been sick for other reasons, in 2003 and 2004.

**Results:**

Women selected a therapeutic option based on perceptions of the severity of the child's condition, confidence in the particular modality, service or practitioner, and affordability of the therapy. While they knew what type of health care was required, poverty in combination with limited availability and perceptions of the poor quality of care at village health centers and public referral hospitals deterred them from doing so. Women initially used home remedies, then sought advice from public and private providers, shifting from one sector to another in a pragmatic response to the child's illness.

**Conclusion:**

The lack of availability of financial resources for poor people and their continuing lack of confidence in the care provided by government centres combine to delay help seeking and inappropriate treatment of children sick with dengue.

## Background

Biotechnical, medical and public health advances have had major impact on the epidemiology and control of infectious disease worldwide. However, the translation of knowledge to practice has been uneven. Vector borne diseases have been particularly intransigent because of the complex interactions of environmental, biological and social factors, and because their prevalence is greatest in poor communities and nations that are least able to respond efficiently to epidemics and to implement effective control strategies. In recent years there has been global political commitment and financial resources to increase the control of malaria and tuberculosis as well as HIV. Other infectious diseases, however, often referred to as tropical diseases but increasingly as neglected diseases of poverty, have received far fewer funds internationally and locally, and are ranked low in priority by local populations and their governments. Limited funds available for and the questionable effectiveness of various biological and chemical interventions have lead to continued emphasis on early diagnosis and treatment of many of these diseases to avert severe morbidity, reduce mortality, and break cycles of transmission and re-infection. The success of this approach depends on cultural, social, economic and institutional factors. Dengue is one such infectious disease that, for various ecological, economic and political reasons, has proved difficult to control.

As dengue hemorrhagic fever (DHF) or dengue shock syndrome (DSS), dengue fever (DF) results in severe morbidity and mortality particularly in young children. Military-style mass interventions in the 1950s gave the impression of its easy control, but subsequently, domestic interventions have had variable success and have rarely been sustainable. Globally, dengue has re-emerged with climate change, rapidly urbanizing areas, poor environmental management and population mobility, as well as the resilience of both the vector (Aedes mosquitos) and the virus [1-4]. In very poor countries where dengue is prevalent, the limited capacity of infectious disease control programs and public health, water and sanitation services, crowded and poor quality housing, and the poor maintenance of public space, contribute to an ideal ecology for the vector to breed and larvae to survive. Most of the social science research on dengue has been concerned with household and community-based interventions to reduce the risk of transmission of disease associated with these environmental factors [5-9]. The emerging evidence is of numerous difficulties associated with suitable methods of communication and sustained community-based vector control [10-12]. Where public interventions with community participation are also problematic, including as a result of conflict and the erosion of security, the one intervention likely to impact on the transmission and severity of infection, and on lowering mortality, is early diagnosis and treatment. Given this, research on social, economic and cultural factors influencing household management of disease, treatment seeking and adherence to advice is vitally important. In order to develop appropriate, acceptable and sustainable interventions, we need to understand far better people's knowledge of the etiology, diagnosis, treatment and impact of various illnesses, and their capacity to act upon such information in ways that protect their own and their families' health. Difficulty in recognizing and differentiating signs of infection of many infectious diseases, and of dangerous deterioration following failure to intervene early, are common factors contributing to infant and child mortality.

In this article, we explore the factors that influence early diagnosis and treatment in Cambodia, focusing on the health seeking behaviour of women with children with suspected dengue fever. As a prelude, we summarize the literature concerned with factors that determine the recognition of illness, household decision-making and treatment seeking. Little of this work has been concerned with dengue fever [13]; the social research literature on infectious diseases of poverty predominantly attends to malaria [14,15]. In the case of malaria, mothers and other caregivers frequently assume that fever is 'normal' or a minor ailment in infants and children [16-19], minimize the (potential) seriousness of fever depending on season [20], and fail to associate related symptoms such as convulsions with malaria [21]. As several authors note [22-25], malaria, dengue, acute respiratory infection, measles, and various other infections all present with fever, leading to confusion regarding diagnosis and severity of the condition, and delays in treatment.

At the outset of symptoms of infection, on the assumption that the condition may be mild, self-limiting and responsive to or relieved by local herbs or simple medication, people typically use home remedies and over-the-counter drugs. Research conducted in Africa in relation to malaria, for example, documents how women treat children with herbs, chloroquine and antipyretics, bought in small quantities from drug stores or small-scale informal traders or saved from previous prescriptions; only when the first measures are ineffective or if the condition of the child deteriorates, do they then present to a local health centre (HC) or a private health provider for advice and assistance [24,26,27]. In Tanzania, the ready availability of antimalarial medicines from small shops, public and private health facilities enables women to provide symptomatic treatment [28], but here as elsewhere, treatment is often inappropriate and the mortality rate remains high from delays in seeking care and the failure of caregivers to complete treatment regimens [29-31]. The inappropriate use of drugs and delays in presenting for biomedical diagnosis and treatment contribute also to continued severe morbidity and mortality from respiratory illness and diarrhoeal disease, as documented in diverse settings [25,32,33]. In the Philippines, for example, women commonly treat children with cough with local herbs and other home remedies, supplemented by over-the-counter antipyretics, cough syrups and other drugs before they seek medical advice [34-36].

These behavioural factors, reflecting local knowledge systems and maternal understandings of children's illnesses, influence health outcomes. But other economic, structural and institutional factors contribute to delays in treatment seeking. These include access to health facilities, quality of care and time costs, as well as costs of transportation and the ability (or not) to pay for prescribed medication as well as the consultation. Poverty is a key factor in influencing whether or not people can meet the direct and indirect costs of treatment. Even with good access to health services, as is the case in Kerala, India [37], the general trend in low and middle income countries for infectious disease and for other health emergencies, such as obstetric care, is that in contrast with wealthier households, poorer households use health services less frequently, delay seeking medical advice, and fail to adhere to treatment recommendations [38-40]. The trend is sharpest in very poor communities and countries, where cash resources are limited and poor quality of care discourages treatment seeking. In this article, using data from rural Cambodia, we illustrate how household poverty and other contextual factors shape pathways to care and the management of disease.

## Methods

Data presented in this article derive from an ethnographic study conducted by the first author from March 2003 to February 2004 in Kampong Cham Province (KCP), eastern Cambodia. Despite the activities of the National Dengue Control Program (NDCP), this province had the highest prevalence of dengue nation-wide. Research was conducted primarily in two villages, Khun and Nekry (pseudonyms), located approximately 30 km in opposite directions of the provincial town centre, Kampong Cham (KPC), around 100 km from the capital city Phnom Penh. Most residents in the study villages, like others throughout the province, were poor farmers who grew rice for subsistence and sale, and who, to meet daily household needs, supplemented rice production by purchasing and reselling other agricultural produce and non-food goods brought from KPC or Phnom Penh. By concentrating the research on two villages, it was possible to collect data for a full calendar year, and so monitor changes in disease prevalence, rainfall, and competing demands on villagers' time according to the agricultural calendar. The time frame also provided sufficient time for the first author to get to know villagers well enough to witness episodes of illness and care seeking, and to gain personal information that would not have been possible in a cross-sectional or otherwise briefer study.

Qualitative methods to collect data included key informant interviews, focus group discussions, in-depth interviews and open-ended questionnaires, and ongoing observations. Focus group discussions and extensive individual interviews were conducted with 28 women from the two villages, whose children had been diagnosed with DF by a local private health worker or had been admitted and diagnosed with DF in KPC Referral Hospital in 2003 or 2004. Group discussions centred on their understanding of the etiology and transmission of dengue, and general patterns of care in the village; interviews gathered details of personal experiences of dengue infection in children and health seeking behaviours. These data were supplemented by in-depth interviews with 38 women, from 19 families per village (approximately 15% of the population) whose children had no history of dengue infection but had been ill for other reasons during the same study period. All interviews on the care of sick children were with women, as all children were cared for by their mothers and other female kin (e.g. grandmother). Key informant interviews were conducted with the village health volunteers in both villages about their perceptions of villagers' awareness of dengue and various health seeking behaviours, and with managers and staff of village HCs, and the provincial and national offices of the dengue control program (N = 15), on dengue case management, health seeking behaviours, and the implications on health seeking of user-fees at public health facilities. Quantifiable entomological and demographic data were also gathered from all households in the study village, and these data enabled us to interpret risk factors for infection using standardised criteria (household index, Breteau index).

All interviews and focus groups were recorded. Recordings were transcribed and translated to English, and analysed using word-processing search functions to enable sequential, contextual and thematic analysis [41-43]. In this process, interviews were analysed line by line to identify specific concepts, events and actors, and these themes were grouped and refined through an iterative process, that included analysis as data were collected and entered, during an extended period after data collection, and again for the preparation of this article. Quantifiable data from interviews as well as from the entomological and environmental observations were entered into Excel spreadsheets for import into SPSS, to enable descriptive analyses using SPSS statistical software. Ethics approval was provided by the University of Melbourne, WHO/TDR, and the Ministry of Health, Cambodia.

## Results

### Treatment choices

Until 1995, health care in Cambodia was provided free of charge. Despite this, local services were underutilized, including because of shortages of essential drugs and other supplies, limited opening and extended waiting times, and pressure on patients to make under-counter payments. Instead of presenting to government health posts, therefore, Khmer villagers routinely used home remedies for health conditions that they considered to be minor. These remedies, still used widely, include herbs gathered from the forest, massage, cupping and 'coining.' The latter term is used to describe a process whereby the caregiver rubs or scratches the back, neck, upper chest, and arms of the sick person with the edge of a coin, often with Tiger Balm™ or other proprietary topical unguent. The area subject to coining becomes grazed, striated and bleeding, and burning from the rubefacient. The redness of the area, and the burning sensations, are seen as evidence that 'wind illness' has been countered, by improving blood circulation and restoring humoural balance. Depending on symptoms, children might be taken to and treated by a traditional healer (*kruu khmer*) with local herbs[44], and/or they may be given over-the-counter drugs, such as paracetamol, purchased at a local outlet or kept at home from a previous episode of illness. If the fever did not resolve, then the child might be taken to a private medical professional prior to seeking care from the public sector [45]. These patterns of treatment and help seeking operate for all childhood illness, and as we illustrate, influence the patterns of care for children diagnosed with dengue.

In 1995, the health system in Cambodia was reorganized to improve the links between community health centres and district hospitals, with centres and hospitals distributed according to population-based criteria. The centres in Khun and Nekry were established in 1998, and like other HCs countrywide, each covered areas of around ten kilometres radius [46]. As part of the reforms, user fees were introduced to generate income to meet short falls in the national budget to sustain the service, to supplement salaries to motivate staff, and to discourage unofficial payments from patients to providers. User fees at the Provincial Referral Hospital, KPC, varied according to service and length of stay: hospitalization from 1 to 4 days cost 30,000 riels (at time of study US$1 = 4000 riels); normal delivery and hospitalization after delivery, 50,000 riels; abortions later than 3 months gestation, or major surgery with laboratory tests, around 200,000 riels. At the local HCs, fees varied from 500 riels for a simple consultation or the provision of oral contraception or condoms, to 15,000 riels for delivery. In the following sections, we describe the home management of fever and other illness, then examine the pathways to care for illness episodes diagnosed as dengue, and examine the wider context and patterns of help seeking.

In order to establish general patterns of treatment seeking in the study area, 38 women whose children had not had dengue in 2003–2004 were asked to explain how they had treated other illness in their children. Consistent with findings from the Cambodia Demographic and Health Survey (DHS) (Cambodia 2000), most respondents had used home treatment as their first action. Eight women had taken no action. Of the remaining women, two thirds administered drugs either already available in the house (e.g. from a previous purchase) or bought over-the-counter from a village store, or used traditional therapies such as massage, coining and herbs in an effort to relieve symptoms. Of the other women, half used village health services – equally presenting to the government health centre or to private practitioners – and half went to KPC either to a private clinic or the provincial referral hospital.

When this first action was not effective, the majority of women sought advice from a health facility, with over 50% seeking advice from a private clinic or government hospital in KPC or Phnom Penh, and one-third attending the health clinic or a private practitioner in the village. Six women, however, turned to traditional therapies and practices, including seeing a fortune teller (*kruu tiey*), a monk (*prah sorng*) or a traditional healer (*kruu khmer*) and using over-the-counter drugs and indigenous herbs to alleviate symptoms. When this second attempt failed to resolve the illness, one-third of women then sought health care from the provincial referral hospital, a private health provider in KPC, or a hospital in Phnom Penh; one third sought advice from the local health centre or a village based private medical practitioner; and one third resorted to prayer, over-the-counter medicines and herbal therapy. In general, women moved between private and public sector health services, and between medical and lay modalities, in an effort to resolve the child's symptoms. Their decisions were informed by a combination of factors, including the diagnosis and understandings of appropriate treatment for symptoms, evaluation of the capacity of health services to assist in treatment, and the accessibility and affordability of various modalities and services. At the same time, although in interviews women spoke of first, second and third actions in the face of unremitting symptoms, in practice women routinely combined therapies to resolve the illness as quickly as possible. Treatment seeking, symptom alleviation and strategies of resort were consequently complicated and often unpredictable.

**Figure 1:**
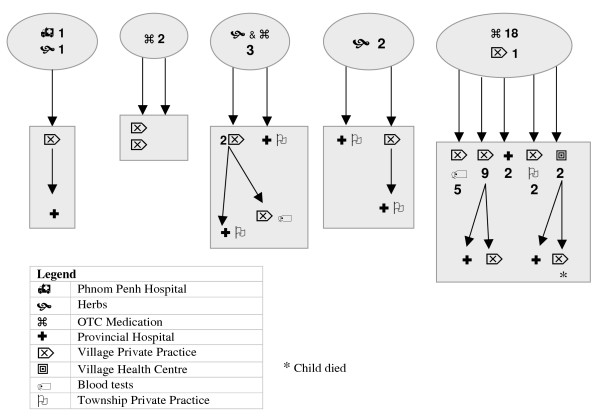
Patterns of help seeking by women with children with dengue.

These patterns of treatment and help-seeking were replicated for children who were diagnosed subsequently with dengue fever – *krun chiem *(*krun *= fever; *chiem *= blood) – as illustrated in Figure 1. The majority of women used drugs purchased over the counter (18/28) as the first course of treatment of their child's illness, at times borrowing money for this purpose: "I buy drugs in the village to treat coughs, cold, fever, a little at a time ... I cant go to the health centre because I'm too busy to wait for the health workers, even if I were to go to the health centre at 7 o'clock in the morning I'd be late for farming ... there's no-one else to do the work." Few women used only traditional home practices such as herbs, cold presses or coining, although five women used pharmaceuticals and traditional therapies concurrently. One child was taken to Kantha Bopha Hospital in Phnom Penh as the first action, not because of perceptions of appropriate care, however, but because the parents were working in a garment factory in the city when the child became sick.

When the child failed to respond to this treatment, women visited a private clinic or arranged for a home visit from a private practitioner because of the convenience of this option in terms of proximity of practitioner and the ability to pay later. One-third of the children visited at home were then taken by the mother to KPC for a blood test to support the clinical diagnosis of DF; the remainder were diagnosed with DF on the basis of clinical signs and a positive tourniquet test, performed by a local practitioner with an elastic cuff. Women believed that local health workers were only able to treat mild illness, and the hours of services and waiting times were "inconvenient." Only two women presented to the local health centre: "they provide services during working hours, but they don't show up even at 8 o'clock. If we had a cut, we'd wait until we stopped bleeding, and still they wouldn't have turned up." Three women went straight to the referral hospital in KPC as their second and last resort of health care. Two women had had negative experiences with the referral hospital previously, however, and they presented instead at a private health practice in KPC. Both private and public heath facilities in KPC offered injections with serum perfusions as the primary treatment. Although health practitioners considered that injections would have limited effect on the treatment of DF, they believed that injections would keep their clients happy and they were able to charge more by doing so.

When children failed to respond to treatment prescribed by local private practitioners, women turned to the referral hospital or private practices in KPC as the next point for care. While three women had taken their child to the referral hospital as the second action, an additional five women used the referral hospital as their third action. Other women presented to local health practitioners and the village HC. Householders without money or property with which to secure a loan to pay for health care again resorted to prayer, home remedies and locally available drugs, purchasing them in the quantity they could afford. Hence women moved between sectors and sites of care according to circumstance, including availability, accessibility and affordability of service, as one women explained:

*"At the beginning, she had a mild fever... I did not know what it was and did not know it was DF...I bought para (Paracetamol) for her, then I felt her...she was cold and it was on the second or third day and she did not talk......I took her to the HC and told them about her condition and told them she was always sleepy. They gave me enough tablets for a day. The next day she became worse and was always sleeping. I could not take her for a blood test and I did not know what to do. I bought medicine locally and kept her until she died. At the time, I had no money, could not do anything and worried about everything. When my child was very sick, I decided to take her for serum perfusion at (the private health practice). They said it was too late...the blood had become clotted, so they couldn't give a perfusion. It was my fault that I hadn't taken her for serum perfusion earlier. ... I was lost ... my husband was busy clearing the land for farming. My daughter was dead when I carried her back home. This is because I had no money. When there is no money, people can't do anything... When my child was sick I took her to the HC, there was no blood test there. If I had taken her for the blood test, then they would have identified DF and she would have got a serum perfusion and she would have been fine.*"

### Village choices

Health staff at the government health centres in the two study villages argued that HC utilization had increased with the reforms, that the services were better than and more accessible than private ones (despite their own dual role, see below), and that the drugs at the HC were more effective than those purchased from provision shops or private practitioners. Villagers, however, had different ideas about the quality of services at these facilities for several reasons. First, as indicated above, while officially the health centres had morning, afternoon and night shifts, health workers usually worked only from around 8 am to 11 am, and were rarely on duty in the afternoon and never at night. Clinic hours were too short for all clients to be seen, clients were reluctant to wait hours without certainty of attention, and they saw little point in going to the HC in the afternoon or night when staff were not on duty and when, if urgent care were needed, they would have to wait for the provider to be called by walkie-talkie or phone. Observations corroborated interviews on the lack of availability and accessibility of clinic staff, suggesting that there had been little change despite health reform.

Villagers in outlying areas served by the HCs faced particular difficulty reaching the clinics in the rainy season when dengue transmission was high and access difficult because of floods and muddy roads. When access was possible, the return trip by motor-bike taxi cost 1000 riels (0.25 USD), beyond the capacity of many families, and consequently, people used available cash to purchase drugs over-the-counter rather than transport. In addition, user fees were a major financial obstacle to seeking care. Upfront payment was required when people presented to HCs, unless they were exempted from the fees. Exemption was relatively arbitrary, however, granted when there was sympathy from a staff member or personal connection to the client. Many people had neither ready cash nor valuables as security for a loan, nor were they able to gain exemption, and therefore could not afford the costs of medical care.

Villagers recognized fever by feeling the bodies of their children, and treated high fever as a sign of possible dengue fever. Women described their children's body temperature as "on fire." They understood that blood testing was the most reliable means by which to confirm dengue, and since they knew that the village HCs lacked the facilities for blood tests, they considered presenting to the HC as simply delaying diagnosis and timely treatment. In the absence of blood testing facilities to distinguish dengue from other febrile infections such as measles, typhoid fever and malaria, HC staff diagnosed on the basis of clinical signs and symptoms: high fever of sudden onset plus at least two of the following: headache, pain in joints or muscles, skin rash and signs of bleeding. From the point of view of villagers, HC staff "just give drugs by guessing." Both HC staff and private health providers usually supplemented clinical observations with a tourniquet test to diagnose, treating the occurrence of petechiae as indicative of dengue infection. In Khun and Nekry HCs, there were no blood pressure cuffs for tourniquet tests. Instead health workers used elastic cuffs to perform the test, but were unfamiliar with the differences in results from the two cuffs, the timing or correct counting of petechiae.

Villagers also questioned the quality and availability of drugs at the HC. HC staff insisted that the quality of HC drugs was better, less expensive and available in greater quantities than at local commercial outlets. However, many villagers believed that the drugs provided by the HCs were effective only for mild disease. Some drugs, usually antipyretics, were included in the consultation fee of 500 riels, compared to 2000 riels for a few tablets for fever, cough or diarrhoea from a village store, but villagers tended to regarded the more expensive over-the-counter drugs to be of higher quality.

Because of the limited opening hours of the clinics and hence poor access to clinic staff, villagers sought health care elsewhere. HC staff often operated private practices from their homes as a second source of income. Charges for private care were fairly standard in the study area and in neighbouring villages: a consultation fee, with medicines for 2–3 days, was approximately 2,000 riels, whether the villager presented to the health provider's home or whether he or she requested a home visit. The most expensive services were for injections and serum perfusions. Whenever the local health practitioner suspected a child of having DF, irrespective of its severity, a serum perfusion would be administered because of parents' beliefs about its efficacy to reduce fever and cure disease, and to compensate for possible dehydration. Villagers saw the perfusion also as a means to make money: they referred to private practitioners as "businessman," providing private care only to those with the money to pay for it.

Even though the same people provided both services and private care was more expensive, private health providers were considered a viable alternative to the HC because the services were always available, home visits were possible and the clinics were located close to other villagers' homes. Although the fees were high, people could pay by instalment, and they sought to do so promptly to establish a good credit rating and ensure the option to pay by instalment again if need be. As in the HC, private providers lacked the equipment for a definitive diagnosis, and so tended to diagnose DF again on the basis of clinical signs and a positive tourniquet test using an elastic cuff. Private providers lacked the equipment to intervene in case of dehydrated shock, and would not give serum perfusion if they thought the child was in shock; instead they would advise the woman to take the child for a blood test in KPC township. Some women did so, however, of their own accord after consulting with relatives or neighbours. Most villagers complained that the care offered by private providers was rarely effective for dengue fever and ultimately they would need to take the child to KPC or Phnom Penh for further treatment. From their perspective, the use of local health care delayed presenting the child to a better health facility and wasted limited financial resources.

### Beyond the village

While these treatment actions were the same as those for any other childhood illness, some women were able to distinguish DF, usually because another child in the household had previously been infected and in a few cases had died, and they were mindful of the need for a blood test to identify the virus and determine appropriate treatment. Other women, even if unclear about DF, could recognize dehydrated shock by the child's cold extremities, high fever and abdominal pain, and this too propelled them to seek help. If health care provided in the village were ineffective, and if the child's parent or other caregiver had the financial resources, then he or she could choose between private and public health services in KPC or Phnom Penh. Villagers knew that public care cost less than private care, but many preferred to use a private provider to avoid the upfront payments required by the Provincial Referral Hospital and again, to avoid long waiting times: "to wait and wait and wait, and come back with empty hands." Because they had no time to sell possessions such as pigs, farm cows, land or their house to realize cash, or to get a loan in the village, they would have difficulty getting attention for the child, as one woman recalled: "I could not take the child to the hospital ... I did not have the money to pay upfront." Apart from the scheduled fees, most interviewees reported having to meet additional charges such as bed or laboratory fees, the cost of certain medicines, catheters and/or blood, depending on hospital policy and availability. These additional costs significantly increased the financial burden for parents, deterring them from utilizing this facility. The alternative was to take the child to a children's hospital in Phnom Penh or Siem Reap, which was managed by a charitable foundation and charged no fees. But travel from KPC to Phnom Penh took approximately two hours and a half by car, and villagers had to find the funds for travel and wait for the service, with emergency cases taking priority. In consequence, both the time costs and direct and indirect cash expenses were high. Many people therefore were forced to keep their child at home, using any home remedy possible, praying and waiting to see the outcome.

Those with access to resources could present to a private health clinic in KPC, run by qualified medical doctors and medical assistants who also worked for the provincial referral hospital, the provincial health department or a town HC. These private clinics varied in size, some operating from people's homes, others located within a purpose-specific house or building. As noted, people preferred these private services because they believed that the quality of care was superior. They could also pay the fee while the child was an inpatient, or could pay after discharge; this gave them some time to secure a loan, sell livestock or mortgage land. Costs were often catastrophic, leading women to compare DF to a house fire or the sinking of their boat, the worst disasters they could imagine in their daily life: "Having DF is like a fire in my house. I have lost a lot of my money... I sold my cows for the treatment, and I am still in debt. I was so scared of this disease."

## Discussion and conclusion

Women identified their child's illness on the basis of physical conditions and clinical signs of DF. All women could describe what affected their children, and could describe fevers that failed to respond to an antipyretic such as paracetamol. They knew what type of health care might be appropriate for effective treatment, that the child would need a blood test to confirm the diagnosis, and that the village HCs lacked the capacity to do this. As a result, provided they had sufficient resources to begin the journey of help seeking, women by-passed the HC and sought care in KPC town or, rarely, in Phnom Penh. For example, one mother took her child to the HC as the first action, then used a cold press and herbal drinks to attempt to reduce the fever, then took the child to the referral hospital in KPC because she was confident neither in the capacity of local staff nor the quality of locally available drugs. But poverty, suspicions of the quality of public health care, and the lack of cash to meet the immediate costs of referral hospitals or the higher, deferred costs from private services, deterred many from doing so immediately. Women consequently used various local treatment options, in varying combinations, while monitoring the child's condition, on the assumption that the child's condition was mild and that outside help other than advice from elderly household members and neighbours was not warranted. Women waited to see if the child's condition would improve and to evaluate the effectiveness of home treatments which, except for medication, involved no cost. Women did not persist with initial home remedies if these failed to bring relief, but would then buy drugs in small quantities and consulted with private practitioners, paying for the consultation at a later date often by instalment.

In the event of continued illness, after two or three days of care, women went to other practitioners or public health services in the village, the provincial centre or Phnom Penh, shifting from one sector to another, in what was a pragmatic response to the child's illness. Household property was sold or pawned for cash to pay for health care, and most households were in debt to village lenders as a result of health-related loans. Women believed that resorting to several therapies would advance the process of diagnosis and healing; they returned to home care again as the last resort when finances were exhausted. These patterns of treatment, help-seeking and observation did not necessarily deviate between dengue and other illnesses, but if women saw signs that they knew to indicate dengue, then they were propelled to act quickly. As illustrated, women selected therapeutic options based on perceptions of the severity of the child's health condition, their confidence in the particular modality, practitioner or service, and the affordability of the service. Women knew dengue to be severe, and knew of dengue-related deaths, but their ability to act promptly was often severely hampered by their lack of access to resources.

In a relatively early paper, Winch and colleagues illustrated that local classifications of DF with other mild, febrile diseases discouraged women from seeking medical advice [25]. In contrast, as we illustrate here, women were often aware of the possibility of DF and were familiar with at least some signs and symptoms. However, the availability of money and perceptions of quality of care were predominant reasons for their actions and sequences of treatment. More recently, Suarez and colleagues [47] have referred to "individuals' therapeutic itineraries" to describe patterns of care seeking for DF in Colombia. The notion of an itinerary is a useful one to describe the patterns of care seeking. While the metaphor draws attention to the meandering and detours that occur in the care of a sick child, increasing the risk of mortality, it fails to capture also the combinations of therapies and merging of modalities. In Cambodia, the itineraries are contingent and often unplanned, and as we note, the availability of financial resources was an especially significant factor determining choice of service and delays in seeking therapy. In interviews, women recurrently spoke of having no money and of "having no money to wait."

As acknowledged in the literature on health care and poverty in Africa, the poorer the household, the higher the burden of health expenditures [48], and the greater the level of poverty, the higher the mortality rates [49]. Treatment patterns, Filmer [38] argues, is strongly related to poverty, and within as well as between communities, wealthier households are more likely to seek timely medical treatment. This is equally so in rural Cambodia. Women consider health care as a commodity, with a price attached to the service, so the availability of money and the time costs determined the itinerary they chose and the stops along the way. Very poor women, while they recognized the severity of their child's conditions and regarded dengue as serious and potentially fatal, had no option but to treat the child at home or to do so even as an interim measure while raising funds. Women could not take children to public hospitals if they did not have money to pay the service fee upfront, and many went to private practitioners for whose services they could pay by over time. But the immediate and long-term costs of care, and so the financial barriers to seeking care, were substantial. While very poor women are entitled to exemptions, villagers questioned the equitable ways in which this entitlement is granted, a possibility not recognised in the optimistic report of this selective strategy, published in *The Lancet *[50]. A recent study of coping strategies and treatment seeking in Kenya [51] illustrates the heavy impact on households through direct treatment costs and time costs, indicating the need for changes such as the elimination of user fees and the introduction of flexible charging systems. Our data, like that of van Damme and colleagues also in Cambodia [52] , point to the need for similar changes to encourage women to present in a more timely manner to health centres.

But in addition, as we have noted, women's lack of confidence in the public sector, particularly at the village level, was an additional disincentive to present. Women questioned the quality of care, quality and effectiveness of drugs, and the reliability of the service, and were well aware of the shortcomings to diagnosis and treat dengue. These findings are not inconsistent with other studies of treatment seeking, where reluctance to use health services derive from a combination of high costs and poor services [51]. Since in rural Cambodia, the private practitioner to whom people took their sick children was often the same person who worked at the health centre (with drugs provided from the same source), people's lack of confidence in government health centres and hospitals suggests continuing broader concerns about the capacity of the state to provide public services. On the other hand, the decision to by-pass local centres in the event of (possible) dengue highlights the realism of their evaluation and the power of pragmatic action.

## Competing interests

The author(s) declare that they have no competing interests.

## Authors' contributions

SK and LM conceived of the study and collaborated in its design. Data collection was conducted by SK as a component of the work required for his Doctor of Philosophy, and was supervised by LM. The authors collaborated in the analysis and interpretation of data and co-wrote this paper. Both authors read and approved the final manuscript.

## Pre-publication history

The pre-publication history for this paper can be accessed here:


